# Association between Atherogenic Dyslipidemia and Subclinical Myocardial Injury in the General Population

**DOI:** 10.3390/jcm13164946

**Published:** 2024-08-22

**Authors:** Nada S. Elbadawi, Moaze H. Sobih, Mai Z. Soliman, Mohamed A. Mostafa, Richard Kazibwe, Elsayed Z. Soliman

**Affiliations:** 1 Epidemiological Cardiology Research Center (EPICARE), Department of Medicine, Section on Cardiovascular Medicine, Wake Forest University School of Medicine, Winston-Salem, NC 27157, USA; nada.elbadawi@med.helwan.edu.eg (N.S.E.); moaze.hassan@med.helwan.edu.eg (M.H.S.); mmostafa@wakehealth.edu (M.A.M.); 2 Undergraduate Campus, Wake Forest University, Winston-Salem, NC 27109, USA; maisoliman2003@outlook.com; 3 Department of Internal Medicine, Wake Forest University School of Medicine, Winston-Salem, NC 27157, USA; rkazibwe@wakehealth.edu

**Keywords:** NHANES-III, atherogenic dyslipidemia, subclinical myocardial injury

## Abstract

**Background:** Subclinical myocardial injury (SCMI) is associated with an increased risk of poor cardiovascular disease (CVD) outcomes. Understanding the underlying risk factors for SCMI is crucial for the prevention and management of CVD. We hypothesized that atherogenic dyslipidemia, a combination of high triglycerides (TG) and low high-density lipoprotein cholesterol (HDL-C), is associated with an increased risk of SCMI. **Methods**: This analysis from the third National Health and Nutrition Examination Survey (NHANES-III) included 7093 participants (age 59.3 ± 13.4 years, 52.8% women, and 49.4% White) free of CVD. Atherogenic dyslipidemia was defined as TG ≥ 150 mg/dL and HDL-C < 40 mg/dL in men or <50 mg/dL in women. A validated electrocardiographic-based cardiac infarction injury score (CIIS) ≥ 10 was considered positive for SCMI. Multivariable logistic regression analysis was used to examine the association of different combinations of TG and HDL-C groups, including atherogenic dyslipidemia with SCMI. **Results:** About 22.5% (*n* = 1594) of participants had atherogenic dyslipidemia, and 26.3% (*n* = 1862) had SCMI. Compared to participants with normal TG and normal HDL-C, those with atherogenic dyslipidemia had a higher prevalence of SCMI (31.2% vs. 23.9%, *p*-value < 0.001). In a multivariable logistic regression model, atherogenic dyslipidemia was associated with the highest odds of SCMI followed by high TG/normal HDL-C, then low HDL-C/normal TG [OR (95% CI): 131 (1.14, 1.52), 1.13 (0.97, 1.33), and 1.01 (0.86, 1.20), respectively). **Conclusions:** Atherogenic dyslipidemia is associated with a higher risk of SCMI, which highlights the role of nontraditional risk factors in the development of subclinical CVD.

## 1. Introduction

There is a dramatic increase in the prevalence of obesity that has led to a marked elevation in metabolic syndrome characterized by visceral adiposity, insulin resistance, elevated blood pressure (BP), elevated triglycerides (TG), and low levels of high-density lipoprotein cholesterol (HDL-C) [1,2]. The mere diagnosis of the metabolic syndrome appears to confer a substantial additional risk of coronary heart disease (CHD) [3,4,5]. Atherogenic dyslipidemia, characterized primarily by elevated TG, low HDL-C, and accumulation of lipoprotein remnants, is a phenotype associated with increased CVD risk [6]. As an entity or through its individual components (TG or HDL-C), atherogenic dyslipidemia has been linked to an increased risk of atherosclerotic CVD (ASCVD) even in patients with controlled LDL-C [6,7]. Moreover, one of the most important features of atherogenic dyslipidemia is the plasma accumulation of small dense LDL (sdLDL) particles, which is characterized by an increased susceptibility to oxidation. In order for the accumulated LDL particles—especially sdLDL—to become atherogenic, LDL needs to undergo chemical modifications, including desialylation and oxidation. When oxidized LDL (ox-LDL) accumulates in predisposed areas of the arterial wall, it contributes to an increased expression of cell adhesion molecules on endothelial cells [8]. Such oxidative stress has been shown to play a major role in the pathogenesis of atherosclerosis [9].

Subclinical myocardial injury (SCMI) refers to early cardiac damage that lacks clear manifestations of CHD. It is identified by an electrocardiographic-based scoring system called the cardiac infarction/injury score (CIIS) exceeding 10 score points. SCMI has been associated with an increased risk of CVD and all-cause mortality [10,11]. Previous studies have linked SCMI with risk factors such as obesity and dyslipidemia [12,13]. However, it is unclear whether atherogenic dyslipidemia is a risk factor for SCMI. We hypothesize that atherogenic dyslipidemia is associated with an increased prevalence of SCMI independent of traditional CVD risk factors, lifestyle factors, and socioeconomic status. We tested this hypothesis in the third National Health and Nutrition Examination Survey (NHANES-III).

## 2. Materials and Methods

### 2.1. Study Population

NHANES, a recurring survey carried out by the National Center for Health Statistics (NCHS) under the Centers for Disease Control and Prevention (CDC), aims to evaluate the health and disease patterns among non-institutionalized civilians in the United States. NHANES-III, conducted from 1988 to 1994, received approval from the NCHS Research Ethics Review Board. All participants provided documented informed consent. Detailed information regarding the study design and methods has been previously disseminated [14].

Per the NHANES-III protocol, only participants 40 years of age and older underwent an electrocardiogram (ECG) recording (*n* = 8561), whom we considered in this analysis, along with those with complete data on atherogenic dyslipidemia. We excluded participants with a prior history of CVD, including ECG evidence of MI or missing key covariates. After all exclusions, a total of 7093 participants were included in the analysis.

### 2.2. Electrocardiographic Subclinical Myocardial Injury

A resting 12-lead ECG was acquired using a Marquette MAC 12 electrocardiograph (Marquette Medical Systems, Milwaukee, WI, USA) during a physical examination conducted in a mobile examination center (MEC). The ECG tracings were automatically processed at the Epidemiological Cardiology Research Center (EPICARE Center, Wake Forest School of Medicine, Winston-Salem, NC, USA) after visual inspection by skilled technicians.

The methods for measuring Cardiac Infarction/Injury Score (CIIS) have been detailed in prior literature [10]. CIIS is a risk-stratified scoring system related to myocardial injury and ischemia, both discrete and continuous, based on a weighted scoring system taking several objective electrocardiographic waveform components. The score is defined by a combination of 11 discrete and 4 continuous features and provides a simple scoring scheme suitable for both visual and computer classification of a standard 12-lead ECG. In the NHANES-III dataset, CIIS values underwent an initial multiplication by a factor of 10 to circumvent the use of decimal points. However, for the current analysis, CIIS values were divided by 10 to present them in their original scale. Similar to prior studies, SCMI was defined as CIIS values equal to or exceeding 10 score points [11].

### 2.3. Atherogenic Dyslipidemia

A phlebotomist collected blood samples via venipuncture. The samples were analyzed for total HDL-C, TG, glucose, and other components in the metabolic panel using laboratory procedures as reported by the National Center for Health Statistics [14]. LDL-C was calculated using the Friedewald equation [15]. Based on the levels of HDL-C and TG, participants were grouped into the following groups: Atherogenic dyslipidemia, defined as high TG (≥150 mg/dL) and low HDL-C (<40 mg/dL in men or <50 mg/dL in women) [16]; high TG/normal HDL-C group; low HDL-C/normal TG; and normal HDL-C/normal TG group.

### 2.4. Covariates

Demographics (age, sex, race) and smoking status were self-reported during an in-home interview. Obesity was defined as body mass index (BMI) ≥ 30 kg/m^2^. Hypertension was defined as systolic BP ≥ 130 mmHg, diastolic BP ≥ 80 mmHg, or the use of antihypertensive medications. Diabetes was defined as fasting blood glucose levels ≥126 mg/dL or the use of glucose-lowering medications. Physical activity was assessed based on the frequency of leisure time activity and included information on types of activity, frequency, and level of activity.

### 2.5. Statistical Analysis

The baseline characteristics were assessed according to atherogenic dyslipidemia status using analysis of variance for continuous variables and chi-square test for categorical variables. Continuous variables were reported as mean and standard deviation (SD) and number and percentage (%) for categorical variables. The normality of the data distribution was assessed using both graphical methods and statistical testing. Specifically, histograms with normal curves, Q–Q plots, and kernel density plots were generated using the SAS ‘PROC SGPLOT’ procedure. Additionally, the Kolmogorov–Smirnov test for normality was performed using the SAS ‘PROC UNIVARIATE’ procedure.

Multivariable logistic regression analysis was used to examine the cross-sectional associations of different combinations of TG and HDL-C groups, including atherogenic dyslipidemia with SCMI. The normal HDL-C/normal TG group was used as the reference group. Model 1 was adjusted for age, sex, race/ethnicity. Model 2 was adjusted for variables in model 1 plus diabetes, hypertension, serum creatinine, body mass index, lipid-lowering medications, smoking, and physical activity. In similar models, we also examined the associations of high TG (vs. normal) and low HDL-C (vs. normal) separately with SCMI.

Analysis was conducted using SAS version 9.4 (SAS Institute Inc., Cary, NC, USA). *p*-values less than 0.05 were considered statistically significant.

## 3. Results

After exclusions, 7093 participants (mean age of 59.3 ± 13.4 years, sex 52.8% women and race 49.4% White) were included in the analysis. Approximately a quarter of the sample (*n* = 1594) had prevalent SCMI. The prevalence of atherogenic dyslipidemia was 22.5%, with average LDL-C levels of 136.4 mg/dL and total cholesterol of 222.2 mg/dL. Table 1 shows the characteristics of the study population stratified by different combinations of TG and HDL-C levels. Compared to individuals with a normal lipid profile (Normal HDL-C/Normal TG), participants with atherogenic dyslipidemia were more likely to be women, Mexican Americans, with higher LDL-C, total cholesterol, and BMI, and had a higher prevalence of diabetes.

The prevalence of SCMI was highest among individuals with atherogenic dyslipidemia (31.2%), as shown in Table 2. However, a similar prevalence of SCMI was observed between individuals with normal HDL-C/normal TG and low HDL-C/normal TG (23.9% vs. 23.8%, respectively). In a multivariable logistic regression model adjusted for demographics and potential confounders, atherogenic dyslipidemia was associated with increased odds of SCMI (OR (95% CI): 1.31 (1.14–1.52). Although similar risk patterns were observed in both the low HDL-C/normal TG and normal HDL-C/high TG groups, the results did not reach statistical significance (OR (95% CI): 1.01 (0.86–1.20), 1.13 (0.97–1.33), respectively).

**Figure 1:**
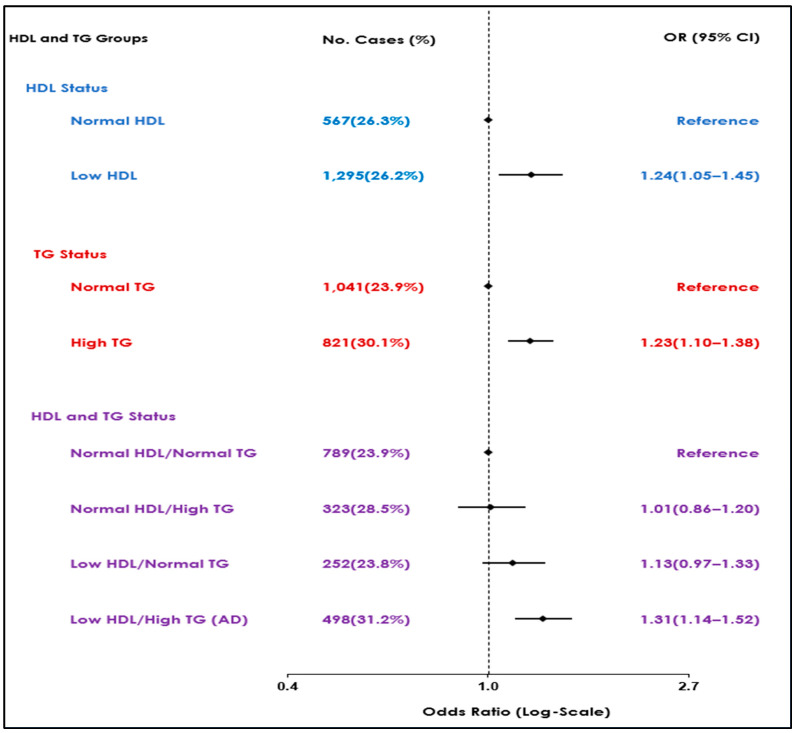
Associations of atherogenic dyslipidemia and different combinations of HDL-C and TG with SCMI.

Further analysis of individual lipid markers utilizing similar models showed increased odds of SCMI in participants with either high TG and low HDL independently compared to normal levels (OR (95% CI): 1.23 (1.10–1.38), 1.24 (1.05–1.45), respectively) (Table 3). Figure 1 summarizes the results of the association observed between lipid markers, either as combinations or individual markers, with the highest risk observed in the atherogenic dyslipidemia group.

## 4. Discussion

Atherogenic dyslipidemia, characterized by abnormalities in the TG–HDL axis, is highly prevalent in patients with CHD, the leading global cause of death. Our analysis from the NHANES-III, a community-based survey, revealed that individuals with atherogenic dyslipidemia had higher odds of SCMI. This association was independent of participants’ demographics or CVD risk factors. Low levels of HDL and higher TG were also independently associated with higher odds of SCMI compared to their normal levels, with a synergistic effect when both abnormalities coexist. These results underscore the role of atherogenic dyslipidemia in the early development of CHD in the form of SCMI and its potential importance in primary prevention and risk stratification.

Except for the atherogenic dyslipidemia pattern, the associations of other different combinations of abnormal TG and HDL with SCMI did not reach statistical significance. As our results showed, low HDL-C compared to normal HDL-C and high TG in comparison to normal TG, separately, were both associated with SCMI, and their strength of association with SCMI was less than that of atherogenic dyslipidemia. This further underscores the augmented effect of the combination posed by atherogenic dyslipidemia phenotype on the process of developing myocardial ischemia.

This effect of high TG and low HDL-C has been linked to an increased risk of ASCVD development and worse cardiovascular outcomes after adjusting for other lipid abnormalities [17,18,19]. Our results suggest that this relationship might have been preceded by subclinical myocardial ischemia and injury before progression to ASCVD.

Hence, the management of atherogenic dyslipidemia could have a bigger role in the prevention of CVD than what is currently thought. This is further underscored by the fact that atherogenic dyslipidemia commonly coexists and correlates with several CVD risk factors [20,21]. It is prevalent in patients with obesity, metabolic syndrome, insulin resistance, and type 2 diabetes mellitus, serving as a marker for increased CVD risk in these populations [6,22,23,24]. The link between atherogenic dyslipidemia and increased CVD risk has also been supported by results from observational studies [7] and clinical trials [6,25].

Atherogenic dyslipidemia is a complex, multifactorial trait, and its contribution to CVD risk is usually estimated through a concomitant presence of hypertriglyceridemia and low HDL-C levels. The association between the atherogenic dyslipidemia phenotype and SCMI can be attributed to interrelated pathophysiological pathways involving metabolic abnormalities such as insulin resistance, genetic factors, oxidative stress, and systemic inflammation.

A hallmark feature of atherogenic dyslipidemia is the accumulation of small dense LDL (sdLDL) particles in the plasma, which are more atherogenic than larger LDL particles due to their higher affinity for endothelial adhesion and susceptibility to lipid peroxidation [26] generates reactive oxygen species (ROS), leading to altered endothelium-dependent vascular relaxation, structural and functional changes, and eventual cell death [27]. These oxidized LDL molecules also increase the expression of molecules vascular cell adhesion molecule-1 (VCAM-1), which are later engulfed by endothelial macrophages creating foam cells [27,28,29].

The imbalance between oxidative stress and the capacity of antioxidants serves as an epitome for early atherosclerosis particularly in the predisposed arterial wall by vascular inflammation [9,28,29,30]. Some studies suggest that oxidative stress is initiated by NADPH oxidase, which generates free radicals that further propagate oxidation, especially in the presence of systemic inflammation [31]. Many ROS are associated with elevated inflammatory biomarkers, such as CRP and interleukins, which are crucial in the interplay between oxidative stress and the pro-inflammatory state linked to metabolic conditions like obesity, insulin resistance, and liver disease [32,33,34].

Insulin resistance is closely tied to qualitative and quantitative lipid modifications, including reduced nuclear expression of lipoprotein lipase, altered function of hormone-sensitive lipase, and increased release of free fatty acids from adipocytes [35,36]. These circulating free fatty acids trigger inflammation by activating Toll-like receptor-2 on cell surfaces, subsequently activating the NF-kB pathway [35]. Activation of this pathway, driven by stimuli such as oxidized LDL and cholesterol crystals, leads to the production of pro-inflammatory cytokines like IL-1β and IL-18 [37]. These cytokines not only promote further recruitment of inflammatory cells but also stimulate the production of IL-6, which increases C-reactive protein levels, which is often associated with SCMI [38]. This chronic, low-grade inflammatory state, in the absence of overt clinical symptoms, eventually lead to subtle disruptions in coronary blood flow and set the stage for progression of overt ASCVD.

Additionally, a hallmark of atherogenic dyslipidemia is an increase in triglyceride-rich lipoproteins in both fasting and postprandial states, which, in circumstances of overproduction or impaired lipolysis and clearance of triglycerides, results in excessive intravascular remodeling and the accumulation of cholesterol remnant in the plasma [39]. The mechanisms conferring increased atherogenicity of remnant lipoproteins include direct deposition of cholesterol into the vascular wall and pro-inflammatory and pro-oxidative properties. It is believed that an increase in postprandial triglycerides may cause endothelial dysfunction through the induction of oxidative stress [40,41]. Additionally, it has been shown that the oxidation of free fatty acids, released during the lipolysis of triglycerides, exerts pro-inflammatory effects [42]. These findings indicate that triglycerides and oxidative stress have interactive roles in atherosclerosis. The complex nature of coronary artery disease, which unfolds over decades and involves both genetic and environmental factors, underscores the importance of genetic predisposition in lipid abnormalities and atherosclerosis. Emerging studies highlight the role of small non-coding microRNAs (miRNAs) in these processes [43,44]. By regulating gene expression at the post-transcriptional level, microRNAs influence lipid metabolism, inflammation, and endothelial function [45]. For example, the presence of miR-122 and miR-370 has been associated with severity of coronary artery disease in patients with dyslipidemia [46]. miR-33a/b have been shown to act as regulators of a lipid metabolism, and their pharmacological inhibition diminished atherosclerosis by raising plasma HDL levels [47]. The inhibition of anti-miR-34a has been showen to reduce vascular inflammation, senescence, and apoptosis, highlighting their prognostic and therapeutic potential [48] Furthermore, genetic variations in the expression of lipoprotein lipase and its associated modulators, such as apoB, have been independently linked to the development of atherosclerosis [45,46]. With these findings in mind, our observed association between atherogenic dyslipidemia and SCMI is most probably due to multiple interacting CVD risk factors and not only the direct effect of atherosclerosis. This is further supported by recent studies showing that coronary artery disease was linked to vascular inflammation independent of coronary artery calcium score, which is a measure of evident atherosclerosis [48,49]. Possible mechanisms include chronic inflammation and the highly atherogenic nature of TG-rich lipoproteins. This is primarily due to their smaller size and high cholesterol content, which facilitates endothelial migration, acting as a substrate for atherosclerosis and myocardial injury [6,49,50,51]. Preventing and controlling atherogenic dyslipidemia will, in turn, prevent excessive cholesterol deposition and oxidation and could lower the risk of SCMI. Lifestyle modification and positive habits targeting regular control of traditional CVD risk factors seem to be promising preventive measures to halt atherogenic dyslipidemia, oxidative stress, and atherosclerosis [8].

It has been suggested that HDL-C levels function more as a biological marker than a therapeutic target for ASCVD [46,52,53]. This is due to the highly heterogeneous nature of HDL particles, in terms of their shape, size, and composition, which arises because of continuous intravascular remodeling in both physiological and pathophysiological states. In atherogenic dyslipidemia, the lipid content of HDL particles is significantly altered and characterized by a decrease in cholesteryl esters with an associated increase in TG content [54]. Moreover, oxidative stress might be an enhancing cause of lipid peroxidation within HDLs, leading to the accumulation of oxidized HDL particles in the plasma of patients with cardiometabolic diseases [55,56]. There is an accompanying pro-inflammatory state that, in turn, alters the protein composition of HDL particles by increasing their serum amyloid A (SAA) content [57]. We showed that lower levels of HDL-C (vs. normal) were associated with an elevated risk of SCMI. These findings reinforce the concept of utilizing low HDL-C levels as an early biomarker for myocardial injury, potentially preceding the manifestation of clinically evident ASCVD. However, the complexity of atherogenic dyslipidemia, with its intertwined components, makes it challenging to isolate and attribute the direct roles of individual components. Although our analysis revealed increased odds of SCMI with induvial components, in real life, these abnormalities rarely occur in isolates, and therapeutic modalities often provide collateral improvement of lipid biomarkers. It is important to take into consideration the influence of other systems on this association. For example, both metabolic-associated fatty liver and non-alcoholic fatty liver disease can help in identifying populations at risk of CVD, which can be attributed to both associated lipid abnormalities or the systematic inflammatory state [58,59].

Although coronary artery disease, as a component of systemic atherosclerosis, is typically seen as a continuously progressing condition, the onset of myocardial ischemia which suggests that an early, identifiable sentinel event in the disease timeline could be pinpointed in this high-risk group. Our study is among the first to demonstrate an association between the atherogenic dyslipidemia phenotype and subclinical myocardial infarction using the CIIS, highlighting the potential rule of incorporating atherogenic dyslipidemia as a whole rather than individual components in risk stratification and supporting the development of more effective preventive strategies. Undoubtedly, the heightened CVD risk associated with the atherogenic dyslipidemia phenotype remains not fully understood. Further research directed towards investigating the pathophysiological effects and the influence of non-LDL-targeting medication on subclinical myocardial injury before they manifest clinically is warranted. Additionally, examining the association in different population samples is needed given the genetic and environmental influences on atherogenic dyslipidemia.

Certain limitations need to be taken into consideration in the interpretation of our study. In this analysis, we only had a single measurement of HDL-C and TG, which may not reflect the status of the long-term lipid profile of the participants. Moreover, we did not incorporate other non-LDL particles (small-dense lipoproteins and lipoprotein remnants) in the diagnosis of atherogenic dyslipidemia, which could undermine the magnitude of the association. Our study design was cross-sectional, and therefore, a causal relationship between atherogenic dyslipidemia and SCMI could not be established. Some of the measurements, like smoking and physical activity, are self-reported and thus subjected to recall bias. Finally, despite adjusting for common CVD risk factors, residual confounding by comorbid conditions that we did not adjust for, e.g., familial hypercholesteremia, remains a possibility. Our study has many strengths as well. This includes a large community-based, multiracial sample size. The ECG and laboratory data were processed in central units. All variables were ascertained using standardized approaches.

## 5. Conclusions

Our results revealed a strong association between AD and SCMI in a racially diverse general population. This underscores the atherogenic effect of this dyslipidemia phenotype and highlights the role of non-traditional risk factors in the development of subclinical CVD. Further research is needed to characterize the pathophysiological mechanisms and explore the influence of lipid management interventions on early myocardial injury.

## Figures and Tables

**Table 1:** Participant characteristics stratified by levels of HDL-C and TG.

Variable	Overall (*n* = 7093)	Normal HDL-C, Normal TG (*n* = 3304)	Normal HDL-C, High TG (*n* = 1134)	Low HDL-C, Normal TG (*n* = 1061)	Atherogenic Dyslipidemia (*n* = 1594)	*p*-Value ^†^
Age, years; mean (SD)	59.3 ± 13.4	59.6 ± 13.8	60.9 ± 12.4	57.3 ± 13.7	59.1 ± 12.8	<0.001
Female; *n* (%)	3743 (52.8)	1762 (23.3)	521 (45.9)	636 (59.9)	824 (51.7)	<0.001
Race-Ethnicity; *n* (%)						<0.001
Non-Hispanic White; *n* (%)	3506 (49.4)	1639 (49.6)	568 (50.1)	480 (45.2)	819 (51.4)	
Non-Hispanic Black; *n* (%)	1588 (22.4)	911 (27.6)	186 (16.4)	277 (26.1)	214 (13.4)	
Mexican-American; *n* (%)	1709 (24.1)	624 (18.9)	350 (30.9)	245 (23.1)	490 (30.7)	
Other; *n* (%)	290 (4.1)	130 (3.9)	30 (2.6)	59 (5.6)	71 (4.5)	
Income <$20k; *n* (%)	3205 (45.2)	1466 (44.4)	532 (46.9)	463 (43.6)	744 (46.7)	0.195
Education ≥High School; *n* (%)	3903 (55.0)	1918 (58.1)	572 (50.4)	611 (57.6)	802 (50.3)	<0.001
Ever Smoker; *n* (%)	3852 (54.3)	1753 (53.1)	640 (56.4)	545 (51.4)	914 (57.3)	0.0032
BMI; mean (SD)	27.6 ± 5.5	26.2 ± 5.1	28.4 ± 4.9	28.6 ± 6.2	29.5 ± 5.4	<0.001
Anti-hypertensive medications; *n* (%)	1543 (21.8)	593 (18.0)	315 (27.8)	210 (19.8)	425 (26.66)	<0.001
LDL, mg/dL; mean (SD)	136.4 ± 38.3	132.7 ± 37.6	142.9 ± 42.9	134.1 ± 34.5	142.2 ± 38.0	<0.001
Lipid Lowering medications; mean (SD)	258 (4.0)	83 (2.5)	67 (5.9)	41 (3.9)	41 (5.9)	<0.001
Total Cholesterol, mg/dL; mean (SD)	222.2 ± 44.2	217.3 ± 40.7	245.8 ± 44.4	197.8 ± 38.4	231.5 ± 44.4	<0.001
SBP, mmHg; mean (SD)	132.9 ± 26.7	131 ± 20.3	137.6 ± 42.9	130.1 ± 30.0	134.5 ± 19.6	<0.001
DBP, mmHg; mean (SD)	76.9 ± 23.9	76.1 ± 17.5	79.6 ± 42.8	76.9 ± 26.9	76.9 ± 10.3	<0.001
Diabetes Mellitus; *n* (%)	1043 (14.7)	301 (9.1)	212 (18.7)	134 (12.6)	396 (24.8)	<0.001
Physically Active; *n* (%)	4797 (67.6)	2323 (10.3)	769 (67.8)	689 (64.9)	1016 (63.4)	<0.001
Serum Creatinine; mean (SD)	1.1 ± 0.4	1.1 ± 0.3	1.1 ± 0.3	1.1 ± 0.6	1.1 ± 0.4	0.027

HDL-C, high-density lipoprotein cholesterol; TG, triglycerides; BMI, body mass index; LDL, low-density lipoprotein cholesterol; SBP, systolic blood pressure; DBP, diastolic blood pressure. Continuous variables are presented as means and standard deviation (SD). Categorical variables are presented as counts and corresponding percentages. ^†^ *p*-values calculated using chi-square test for categorical variables and analysis of variance for continuous variables.

**Table 2:** Association of atherogenic dyslipidemia and other TG/HDL-C combinations with SCMI.

TG/HDL-C Group	No. Event (%)	Model 1		Model 2	
		OR (95% CI)	*p*-Value	OR (95% CI)	*p*-Value
Normal HDL-C, Normal TG	789 (23.9)	Ref	--	Ref.	--
Low HDL-C, Normal TG	252 (23.8)	1.09 (0.92–1.29)	0.320	1.01 (0.86–1.20)	0.877
Normal HDL-C, High TG	323 (28.5)	1.23 (1.06–1.44)	0.008	1.13 (0.97–1.33)	0.120
Atherogenic dyslipidemia	498 (31.2)	1.52 (1.32–1.74)	<0.001	1.31 (1.14–1.52)	<0.001

OR, odds ratio; CI, confidence interval; SCMI, subclinical myocardial injury; HDL-C, high-density lipoprotein cholesterol; TG, triglycerides. Model 1 adjusted for age, sex, race/ethnicity; Model 2 adjusted for model 1 plus diabetes, hypertension, serum creatinine, body mass index, smoking, physical activity, and lipid-lowering medications.

**Table 3:** Association of TG and HDL-C, separately, with SCMI

TG and HDL-C Status	No. Event (%)	Model 1		Model 2	
		OR, CI (95%)	*p*-Value	OR, CI (95%)	*p*-Value
Normal TG	1041 (23.9)	Ref	--	Ref.	--
High TG	821 (30.1)	1.36 (1.22–1.52)	<0.001	1.23 (1.10–1.38)	<0.001
Normal HDL-C	567 (26.3)	Ref	--	Ref	--
Low HDL-C	1295 (26.2)	1.34 (1.14–1.57)	<0.001	1.24 (1.05–1.45)	0.011

OR, odds ratio; CI, confidence interval; SCMI, subclinical myocardial injury. Model 1 adjusted for age, sex, race/ethnicity; Model 2 adjusted for model 1 plus diabetes, hypertension, serum creatinine, body mass index, smoking, physical activity, and lipid-lowering medications.

## Data Availability

The data used in this study are publicly available at the CDC website https://wwwn.cdc.gov/nchs/nhanes/nhanes3/datafiles.aspx, accessed on 6 July 2024.

## References

[1] Ford E.S., Giles W.H., Dietz W.H. (2002). Prevalence of the metabolic syndrome among US adults: Findings from the third National Health and Nutrition Examination Survey. JAMA.

[2] Grundy S.M., Cleeman J.I., Merz C.N., Brewer H.B., Clark L.T., Hunninghake D.B., Pasternak R.C., Smith S.C., Stone N.J. (2004). Implications of recent clinical trials for the National Cholesterol Education Program Adult Treatment Panel III guidelines. Circulation.

[3] Guo Y., Musani S.K., Sims M., Pearson T.A., DeBoer M.D., Gurka M.J. (2018). Assessing the added predictive ability of a metabolic syndrome severity score in predicting incident cardiovascular disease and type 2 diabetes: The Atherosclerosis Risk in Communities Study and Jackson Heart Study. Diabetol. Metab. Syndr..

[4] McNeill A.M., Katz R., Girman C.J., Rosamond W.D., Wagenknecht L.E., Barzilay J.I., Tracy R.P., Savage P.J., Jackson S.A. (2006). Metabolic syndrome and cardiovascular disease in older people: The cardiovascular health study. J. Am. Geriatr. Soc..

[5] Fan W., Philip S., Granowitz C., Toth P.P., Wong N.D. (2020). Prevalence of US Adults with Triglycerides  ≥  150 mg/dL: NHANES 2007–2014. Cardiol. Ther..

[6] Lorenzatti A.J., Toth P.P. (2020). New Perspectives on Atherogenic Dyslipidaemia and Cardiovascular Disease. Eur. Cardiol. Rev..

[7] Valensi P., Avignon A., Sultan A., Chanu B., Nguyen M.T., Cosson E. (2016). Atherogenic dyslipidemia and risk of silent coronary artery disease in asymptomatic patients with type 2 diabetes: A cross-sectional study. Cardiovasc. Diabetol..

[8] Bale B.F., Doneen A.L., Leimgruber P.P., Vigerust D.J. (2022). The critical issue linking lipids and inflammation: Clinical utility of stopping oxidative stress. Front. Cardiovasc. Med..

[9] Khosravi M., Poursaleh A., Ghasempour G., Farhad S., Najafi M. (2019). The effects of oxidative stress on the development of atherosclerosis. Biol. Chem..

[10] Rautaharju P.M., Warren J.W., Jain U., Wolf H.K., Nielsen C.L. (1981). Cardiac infarction injury score: An electrocardiographic coding scheme for ischemic heart disease. Circulation.

[11] O’Neal W.T., Shah A.J., Efird J.T., Rautaharju P.M., Soliman E.Z. (2014). Subclinical myocardial injury identified by cardiac infarction/injury score and the risk of mortality in men and women free of cardiovascular disease. Am. J. Cardiol..

[12] Vasim I., Ahmad M.I., Mongraw-Chaffin M., Soliman E.Z. (2019). Association of Obesity Phenotypes with Electrocardiographic Subclinical Myocardial Injury in the General Population. Clin. Cardiol..

[13] Liu Y., Wu M., Xu J., Sha D., Xu B., Kang L. (2020). Association Between Triglyceride and Glycose (TyG) Index and Subclinical Myocardial Injury. Nutr. Metab. Cardiovasc. Dis..

[14] US Department of Health and Human Services (1994). Plan and Operation of the Third National Health and Nutrition Examination Survey, 1988–1994. Series 1: Programs and collection procedures, No. 32. Vital and Health Statistics.

[15] Friedewald W.T., Levy R.I., Fredrickson D.S. (1972). Estimation of the concentration of low-density lipoprotein cholesterol in plasma, without use of the preparative ultracentrifuge. Clin. Chem..

[16] Busquets-Cortés C., López C., Paublini H., Arroyo Bote S., López-González Á.A., Ramírez-Manent J.I. (2022). Relationship between Atherogenic Dyslipidaemia and Lipid Triad with Different Scales of Overweight and Obesity in 418,343 Spanish Workers. J. Nutr. Metab..

[17] Guyton J.R., Slee A.E., Anderson T., Fleg J.L., Goldberg R.B., Kashyap M.L., Marcovina S.M., Nash S.D., O’Brien K.D., Weintraub W.S. (2013). Relationship of lipoproteins to cardiovascular events: The AIM-HIGH Trial (Atherothrombosis Intervention in Metabolic Syndrome With Low HDL/High Triglycerides and Impact on Global Health Outcomes). J. Am. Coll. Cardiol..

[18] Athyros V.G., Tziomalos K., Karagiannis A., Mikhailidis D.P. (2011). Dyslipidaemia of obesity, metabolic syndrome and type 2 diabetes mellitus: The case for residual risk reduction after statin treatment. Open Cardiovasc. Med. J..

[19] Banegas J.R., Lopez-Garcia E., Dallongeville J., Guallar E., Halcox J.P., Borghi C., Massó-González E.L., Jiménez F.J., Perk J., Steg P.G. (2011). Achievement of treatment goals for primary prevention of cardiovascular disease in clinical practice across Europe: The EURIKA study. Eur. Heart J..

[20] Pappan N., Awosika A.O., Rehman A. (2024). Dyslipidemia. StatPearls.

[21] Manjunath C.N., Rawal J.R., Irani P.M., Madhu K. (2013). Atherogenic dyslipidemia. Indian J. Endocrinol. Metab..

[22] Paublini H., López González A.A., Busquets-Cortés C., Tomas-Gil P., Riutord-Sbert P., Ramírez-Manent J.I. (2023). Relationship between Atherogenic Dyslipidaemia and Lipid Triad and Scales That Assess Insulin Resistance. Nutrients.

[23] Musunuru K. (2010). Atherogenic dyslipidemia: Cardiovascular risk and dietary intervention. Lipids.

[24] Costacou T., Miller R.G., Bornfeldt K.E., Heinecke J.W., Orchard T.J., Vaisar T. (2024). Sex differences in the associations of HDL particle concentration and cholesterol efflux capacity with incident coronary artery disease in type 1 diabetes: The RETRO HDLc cohort study. J. Clin. Lipidol..

[25] Lawler P.R., Akinkuolie A.O., Chu A.Y., Shah S.H., Kraus W.E., Craig D., Padmanabhan L., Glynn R.J., Ridker P.M., Chasman D.I. (2017). Atherogenic Lipoprotein Determinants of Cardiovascular Disease and Residual Risk Among Individuals with Low Low-Density Lipoprotein Cholesterol. J. Am. Heart Assoc..

[26] Krauss M. (2022). Small dense low-density lipoprotein particles: Clinically relevant?. Curr. Opin. Lipidol..

[27] Rizzo M., Berneis K. (2006). Low-density lipoprotein size and cardiovascular risk assessment. QJM.

[28] Berneis K., Krauss M. (2002). Metabolic origin and clinical significance of LDL heterogeneity. Lipid Res..

[29] Rasheed A., Cummins L. (2018). Beyond the Foam Cell: The Role of LXRs in Preventing Atherogenesis. Int. J. Mol. Sci..

[30] Rizzo M., Kotur-Stevuljevic J., Berneis K., Spinas G., Rini G.B., Jelic-Ivanovic Z., Spasojevic-Kalimanovska V., Vekic J. (2009). Atherogenic dyslipidemia and oxidative stress: A new look. Transl. Res..

[31] Abais J.M., Xia M., Zhang Y., Boini K.M., Li P.L. (2015). Redox regulation of NLRP3 inflammasomes: ROS as trigger or effector?. Antioxid. Redox Signal..

[32] Mittal M., Siddiqui M.R., Tran K., Reddy S.P., Malik A.B. (2014). Reactive oxygen species in inflammation and tissue injury. Antioxid. Redox Signal..

[33] Peterson S.J., Shapiro J.I., Thompson E., Singh S., Liu L., Weingarten J.A., O’Hanlon K., Bialczak A., Bhesania S.R., Abraham N.G. (2019). Oxidized HDL, Adipokines, and Endothelial Dysfunction: A Potential Biomarker Profile for Cardiovascular Risk in Women with Obesity. Obesity.

[34] Janac M., Zeljkovic A., Jelic-Ivanovic D., Dimitrijevic-Sreckovic S., Vekic J., Miljkovic M., Stefanovic A., Kotur-Stevuljevic J.M., Ivanisevic J.M., Spasojevic-Kalimanovska V.V. (2020). Increased Oxidized High-Density Lipoprotein/High-Density Lipoprotein-Cholesterol Ratio as a Potential Indicator of Disturbed Metabolic Health in Overweight and Obese Individuals. Lab. Med..

[35] Bobik A. (2008). Apolipoprotein CIII and atherosclerosis: Beyond effects on lipid metabolism. Circulation.

[36] Grandl G., Wolfrum C. (2018). Hemostasis, endothelial stress, inflammation, and the metabolic syndrome. Semin. Immunopathol..

[37] Welty F.K., Alfaddagh A., Elajami T.K. (2016). Targeting inflammation in metabolic syndrome. Transl. Res..

[38] Jin Y., Fu J. (2019). Novel Insights into the NLRP 3 Inflammasome in Atherosclerosis. J. Am. Heart Assoc..

[39] Vekic J., Stromsnes K., Mazzalai S., Zeljkovic A., Rizzo M., Gambini J. (2023). Oxidative Stress, Atherogenic Dyslipidemia, and Cardiovascular Risk. Biomedicines.

[40] Libby P., Ridker P.M., Hansson G.K. (2011). Progress and challenges in translating the biology of atherosclerosis. Nature.

[41] Man A.W.C., Li H., Xia N. (2020). Impact of Lifestyles (Diet and Exercise) on Vascular Health: Oxidative Stress and Endothelial Function. Oxid. Med. Cell. Longev..

[42] Henein M.Y., Vancheri S., Longo G., Vancheri F. (2022). The Role of Inflammation in Cardiovascular Disease. Int. J. Mol. Sci..

[43] Badi I., Mancinelli L., Polizzotto A., Ferri D., Zeni F., Burba I., Milano G., Brambilla F., Saccu C., Bianchi M.E. (2018). miR-34a Promotes Vascular Smooth Muscle Cell Calcification by Downregulating SIRT1 (Sirtuin 1) and Axl (AXL Receptor Tyrosine Kinase). Arterioscler. Thromb. Vasc. Biol..

[44] Raucci A., Macrì F., Castiglione S., Badi I., Vinci M.C., Zuccolo E. (2021). MicroRNA-34a: The bad guy in age-related vascular diseases. Cell. Mol. Life Sci..

[45] Natarajan P., Kohli P., Baber U., Nguyen K.H., Sartori S., Reilly D.F., Mehran R., Muntendam P., Fuster V., Rader D.J. (2015). Association of APOC3 Loss-of-Function Mutations with Plasma Lipids and Subclinical Atherosclerosis: The Multi-Ethnic BioImage Study. J. Am. Coll. Cardiol..

[46] Crosby J., Peloso G.M., Auer P.L., Crosslin D.R., Stitziel N.O., Lange L.A., Lu Y., Tang Z.-Z., Zhang H., TG and HDL Working Group of the Exome Sequencing Project, National Heart, Lung, and Blood Institute (2014). Loss-of-function mutations in APOC3, triglycerides, and coronary disease. N. Engl. J. Med..

[47] Zhan J., Qin S., Lu L., Hu X., Zhou J., Sun Y., Yang J., Liu Y., Wang Z., Tan N. (2016). miR-34a is a common link in both HIV- and antiretroviral therapy-induced vascular aging. Aging.

[48] Gao W., He H.W., Wang Z.M., Zhao H., Lian X.Q., Wang Y.S., Zhu J., Yan J.J., Zhang D.G., Yang Z.J. (2012). Plasma levels of lipometabolism-related miR-122 and miR-370 are increased in patients with hyperlipidemia and associated with coronary artery disease. Lipids Health Dis..

[49] Ono K. (2016). Functions of microRNA-33a/b and microRNA therapeutics. J. Cardiol..

[50] Saydam C.D. (2023). Subclinical cardiovascular disease and utility of coronary artery calcium score. Int. J. Cardiol. Heart Vasc..

[51] Cho I., Suh J.W., Chang H.J., Kim K.I., Jeon E.J., Choi S.I., Cho Y.S., Youn T.J., Chae I.H., Kim C.H. (2013). Prevalence and prognostic implication of non-calcified plaque in asymptomatic population with coronary artery calcium score of zero. Korean Circ. J..

[52] Quispe R., Martin S.S., Michos E.D., Lamba I., Blumenthal R.S., Saeed A., Lima J., Puri R., Nomura S., Tsai M. (2021). Remnant cholesterol predicts cardiovascular disease beyond LDL and ApoB: A primary prevention study. Eur. Heart J..

[53] Varbo A., Benn M., Tybjaerg-Hansen A., Nordestgaard B.G. (2013). Elevated remnant cholesterol causes both low-grade inflammation and ischemic heart disease, whereas elevated low-density lipoprotein cholesterol causes ischemic heart disease without inflammation. Circulation.

[54] von Eckardstein A. (2022). High Density Lipoproteins: Is There a Comeback as a Therapeutic Target?. Handb. Exp. Pharmacol..

[55] Razavi A.C., Mehta A., Jain V., Patel P., Liu C., Patel N., Eisenberg S., Vaccarino V., Isiadinso I., Sperling L.S. (2023). High-Density Lipoprotein Cholesterol in Atherosclerotic Cardiovascular Disease Risk Assessment: Exploring and Explaining the “U”-Shaped Curve. Curr. Cardiol. Rep..

[56] Bartlett J., Predazzi I.M., Williams S.M., Bush W.S., Kim Y., Havas S., Toth P.P., Fazio S., Miller M. (2016). Is isolated low high-density lipoprotein cholesterol a cardiovascular disease risk factor? New insights from the Framingham Offspring Study. Circ. Cardiovasc. Qual. Outcomes.

[57] Girona J., Amigo N., Ibarretxe D., Plana N., Rodriguez-Borjabad C., Heras M., Ferre R., Gil M., Correig X., Masana L. (2019). HDL Triglycerides: A New Marker of Metabolic and Cardiovascular Risk. Int. J. Mol. Sci..

[58] Webb R. (2021). High-Density Lipoproteins and Serum Amyloid A (SAA). Curr. Atheroscler. Rep..

[59] Cheng Y., Wang M., Zheng S., Xia M., Yang H., Zhang D., Yin C., Cheng N., Bai Y. (2022). Comparing the Diagnostic Criteria of MAFLD and NAFLD in the Chinese Population: A Population-based Prospective Cohort Study. J. Clin. Transl. Hepatol..

